# Functional and Anatomical Outcomes of Pars Plana Vitrectomy for Epiretinal Membrane in Patients with Uveitis

**DOI:** 10.3390/diagnostics12123044

**Published:** 2022-12-05

**Authors:** Irina-Elena Cristescu, Tsveta Ivanova, George Moussa, Mariantonia Ferrara, Niall Patton, Felipe Dhawahir-Scala, Soon Wai Ch’ng, Arijit Mitra, Ajai K. Tyagi, Kim Son Lett, Assad Jalil

**Affiliations:** 1 Manchester Royal Eye Hospital, Manchester University Hospitals NHS Foundation Trust, Manchester M13 9WL, UK; 2 Birmingham and Midland Eye Centre, Sandwell and West Birmingham Hospitals NHS Trust, Birmingham B18 7QH, UK

**Keywords:** cystoid macular edema, epiretinal membrane peel, internal limiting membrane peel, ocular coherence tomography, pars plana vitrectomy, uveitis, vitreomacular traction, vitreoretinal

## Abstract

**Purpose-**To evaluate the anatomical and functional outcomes of vitrectomy and epiretinal membrane (ERM) peeling in patients with uveitis. Secondarily, we evaluated the effect of internal limiting membrane (ILM) peeling on surgical outcomes, and of surgery on uveitis activity and, thus, therapeutic regime. **Methods-**Bicentre, retrospective, interventional case series of 29 eyes of 29 consecutive patients affected by uveitis and ERM, that had undergone pars plana vitrectomy with ERM peel between 2012 and 2020, with a minimum postoperative follow-up (FU) of six-months. Demographic data, best-corrected visual-acuity (BCVA), clinical findings, intraoperative and postoperative complications, and macular optical-coherence-tomography scans were reviewed. **Results-**The mean (standard deviation) duration of follow-up was 32 (22) months. At six-month FU, mean central-retinal-thickness (CRT) significantly improved (from 456 (99) to 353 (86) microns; *p* < 0.001), and mean BCVA improved from 0.73 (0.3) to 0.49 (0.36) logMAR (*p* < 0.001), with only one (3%) patient experiencing worsening of vision. The rate of concomitant cystoid macular edema decreased from 19 (66%) eyes at presentation to eight (28%) eyes at final-FU (*p* = 0.003). Comparing eyes in which ILM peeling was performed in addition to ERM peeling only, BCVA or CRT reduction were comparable. Only a minority of six (21%) eyes had a worsening in uveitis activity requiring additional medications, whereas most patients resumed the same treatment (52%) or received less treatment (28%) (*p* = 0.673). **Conclusions-**Vitrectomy with ERM peeling led to favourable anatomical and functional outcomes in patients with uveitis regardless of whether the ILM is peeled or not. As in most patients, no activation of the uveitis requiring additional medications was noted, we do not recommend changes in anti-inflammatory/immunosuppressive therapy postoperatively.

## 1. Introduction

Epiretinal membrane (ERM), with or without cystoid macular edema (CME), is a common complication in eyes with uveitis, with a reported incidence varying from 18 to 69% [1,2]. It is responsible for potentially severe visual impairment, mainly in terms of reduction of visual acuity (VA) and metamorphopsia [3]. The development of optical coherence tomography (OCT) has revolutionised the assessment of ERM and, in general, of diseases of vitreoretinal interface [4]. The possibility to visualize retinal layers at high-resolution contributed significantly to the understanding of the pathogenesis of multiple retinal diseases, including ERM [5]. In addition, multiple findings detectable on OCT have been identified as prognostic factors for visual recovery after surgery [6].

As for idiopathic ERM, pars plana vitrectomy (PPV) with ERM peeling is the procedure of choice for the treatment of uveitic ERM. However, in the latter, several aspects of the surgical approach are still under debate, such as timing, perioperative therapeutic management, or combination of internal limiting membrane (ILM) peeling. Furthermore, due the relative paucity of these patients, the evidence supporting a beneficial effect of PPV for uveitic ERM in improving VA [7,8,9,10] and reducing the recurrences of CME and inflammation [11,12], is still very limited. Hence, we conducted this multicentric study in the second and third largest vitreoretinal centres in the UK, aiming to assess the role of PPV with ERM peeling on anatomical and functional outcomes of patients with uveitis. Specifically, we primarily focused on the evaluation of best-corrected VA (BCVA) and central retinal thickness (CRT); secondarily, we reported on impact of: (i) ILM peeling on BCVA and CRT; (ii) surgical procedure on uveitis activity and, thus, treatment regimen; and (iii) potential factor influencing the outcomes and therapeutic regimen, in particular, for concomitant CME.

## 2. Materials and Methods

### 2.1. Study Design, Participants and Ethics 

We carried out a multicentre, interventional, continuous retrospective study on patients with ERM secondary to uveitis, who underwent PPV and ERM peeling either at Manchester Royal Eye Hospital (MREH) or at Birmingham and Midland Eye Centre (BMEC) from January 2012 to December 2020. We included only patients with a minimum follow-up (FU) of six months. As this was a retrospective anonymized study, as per our local protocol from our Clinical Effectiveness Department, and national guidelines from the National Code of Clinical Research and the Health Research Authority (HRA), this study has ethical approval exemption and no patient consent was required for participation [13,14]. All procedures were completed prior to the design of this study. Indeed, patients and the public were not involved in this study, patients were diagnosed and treated according to local guidelines and agreements and written consent from patients was acquired prior to all procedures as clinically indicated. This study does not report on the use of new or experimental protocols. 

### 2.2. Data Collection

From MREH and BMEC, Microsoft Access and Excel databases, and electronic patient records (EPR, Medisoft Ophthalmology, Medisoft Limited, Leeds, UK) were used to extract data. Data collected include patients’ demographics, aetiology of uveitis, comorbidities, preoperative and postoperative status of uveitis (active/inactive) and treatment regimen, surgical details, postoperative complications, and visual and anatomical outcomes. 

In our units, all patients undergo a comprehensive ophthalmic examination preoperatively (within two weeks before surgery) and at each FU visits, including BCVA evaluation, intraocular pressure measurement through applanation tonometry, slit lamp biomicroscopy, dilated fundus examination, and optical coherence tomography (OCT) of the macula using either Topcon 3d OCT 2000 or Heidelberg SPECTRALIS^®^ HRA + OCT. If needed, fluorescein angiography (FA) was performed to differentiate between residual cystic spaces and exudative macular edema. Anterior chamber inflammation was graded according to SUN criteria [15] and was defined “mild” if AC cells were ≤+1, “moderate” if +2 or +3, and “severe” if +4. Vitreous inflammation was graded following Nussenblatt’s method [16].

Treatment regimen was categorized to (i) no medication, (ii) topical steroid (TS) only, (iii) oral steroid (OS) only, (iv) conventional or biologic Disease Modifying Anti-Rheumatic Drugs (DMARDs), or (v) a combination of them. 

### 2.3. Surgical Technique and Perioperative Treatment

All patients underwent transconjunctival small gauge three-port PPV (23 and 25 gauge) by eight different experienced vitreoretinal surgeons under local anaesthesia. All phakic patients underwent combined phacoemulsification with intraocular lens implantation. After posterior vitreous detachment induction (if not present), core and peripheral vitrectomy, a combined blue dye of trypan blue 0.15% + brilliant blue 0.025% + polyethylene glycol 3350 4% (MEMBRANEBLUE-DUAL^®^ Dye, DORC International, Zuidland, The Netherlands) was used to facilitate membrane peeling. Once ERM was peeled, the peeling of the ILM was performed as per the operating surgeon’s preference. Any retinal breaks found during the indented search were treated with cryotherapy or endolaser retinopexy. Finally, fluid-air exchange was performed. No postoperative posture was advised.

Adjuvant treatments, including injection of triamcinolone acetonide (TA) (Subtenons’ or intravitreal) or perioperative pulsed intravenous methylprednisolone (IVMP), were prescribed based on patient weight, uveitis status, and surgeon preference.

For all patients, the postoperative therapeutic regimen included only topical medications, such as prednisolone acetate 1% every two hours in the first week followed by a gradual tapering (1 drop every week) to preoperative regimen, chloramphenicol 0.5% four times a day for 2 weeks, and cyclopentolate 1% twice a day for 2 weeks.

### 2.4. Statistical Analysis

The statistical analysis was completed with IBM SPSS Statistics software (version 28; Armonk, NY, USA: IBM Corp). Statistical significance was defined as *p* < 0.05. Prior to analysis, continuous variables were assessed using the Shapiro–Wilk test. Hence, non-normally distributed data are reported as medians and interquartile ranges (IQRs), otherwise mean (standard deviation) are reported. For univariate comparisons, the Mann–Whitney U was used to compare two independent variables. Wilcoxon signed rank test was used for two-paired data. Fisher exact test was used for nominal variables. McNemar-Bowker test was used for paired nominal variables. 

The visual acuity was measured on ETDRS charts and converted to logMAR units for 17 of 29 eyes. The remaining 12 values were converted from Snellen to logMAR, applying an acknowledged formula [17,18]. A difference of minimum 0.2 logMAR units (two lines on the EDTRS chars) was recorded as a postoperative visual change. 

## 3. Results

### 3.1. Baseline Characteristics

We included 29 eyes of 29 subjects, of which 14 (48%) males and 15 (52%) females, with a mean (standard deviation) age at the time of surgery of 64 (10) years. The mean documented time between the uveitis onset and the ERM diagnosis was 29 (40) months, and the mean duration of postoperative FU was 32 (22) months. Type and aetiology of uveitis are documented in Table 1. 

At the baseline, eight eyes (28%) were phakic. The uveitis was inactive in the majority the patients (18 (62%)), whereas AC inflammation was detected in eight (28%) eyes and vitritis in three (10%) cases. Concomitant CME was present in 19 eyes (66%) of the eyes.

In terms of baseline therapeutic regimen, 15 (52%) patients were on topical steroids only, four (14%) on combined topical and OS, two (7%) on OS only, and two (7%) on topical and OS and DMARD (one on methotrexate and one on infliximab). 

### 3.2. Surgical Details

Epiretinal membrane peeling alone was performed in 16 (55%) eyes, whereas combined ERM/ILM peeling off in 13 (45%) eyes. Laser or cryo-retinopexy was performed in seven cases (24%). Intraoperative complications were registered in a total of six eyes (21%) and entailed iatrogenic breaks in five (17%) cases and iris prolapse in one case (3%). 

At the end of the surgery, an intravitreal injection of preservative-free TA suspension for intraocular use (Triescence, ALCON LABORATORIES, Fort Worth, TX, USA) was given in six cases (21%), whilst 1 mL of preserved TA 4 mg/0.1 mL (Kenalog, Bristol-Myers Squibb, Madrid, Spain) was administered in form of Subtenons’ injection in one case (3%). Two (7%) cases received pulsed IVMP. 

### 3.3. Visual Outcomes

**Figure 1:**
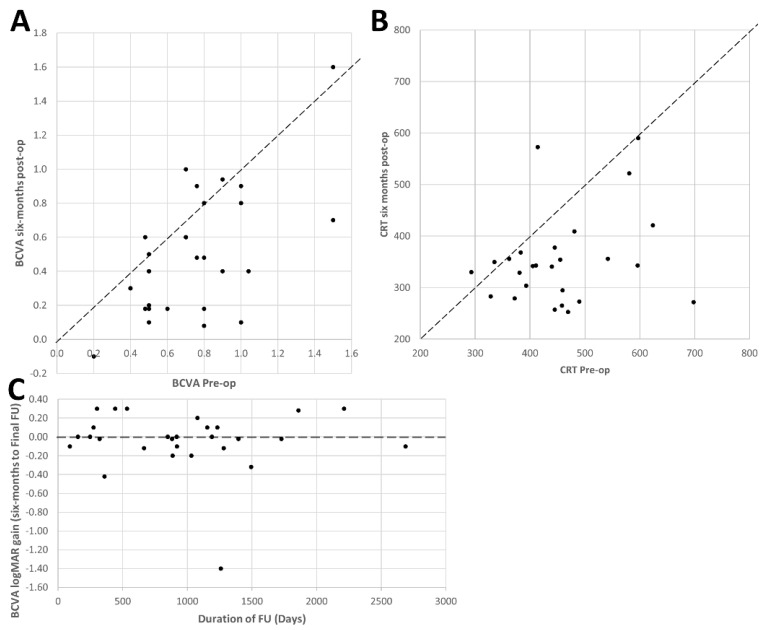
Central retinal thickness and visual acuity outcomes following ERM peel in uveitis patients. CRT: Central Retinal Thickness, ERM: Epiretinal Membrane, BCVA: Best Corrected Visual Acuity., FU: Follow-up. Below the diagonal dashed black line represents improvement postoperatively for (**A**) visual acuity post-operatively (*p* < 0.001) and (**B**) CRT reduced significantly post-operatively (*p* < 0.001). (**C**) Above the dashed black line represents improvement in BCVA at final FU relative to six-months. Even with prolonged FU, patients maintain their post-operative logMAR gain with no difference at six-month and final FU (*p* = 0.726).

Pre-operative BCVA relative to post-operative BCVA at six-months can be found in Figure 1A. 

Relative to the baseline, mean postoperative BCVA improved at six months (*p* < 0.001) (Figure 1A). This improvement in vision was maintained over time with no difference detected between BCVA at six months and final FU (*p* = 0.726) (Figure 1B). Specifically, at 6-month FU, BCVA improved in 15 (52%) eyes, remained stable in 13 (45%), and worsened in 1 case (3%); whereas, at the final FU mean BCVA was improved in 19 eyes (66%), stable in 8 (28%), and worse in 2 eyes (7%). The causes of reduced BCVA were entailed unstable IOL in one patient and uveitic glaucoma leading to final VA of no perception of light in another patient. We demonstrate that over time, patients with long FU had maintained improvement in BCVA compared to six-months FU (Figure 1C) (*p* = 0.726).

**Figure 2:**
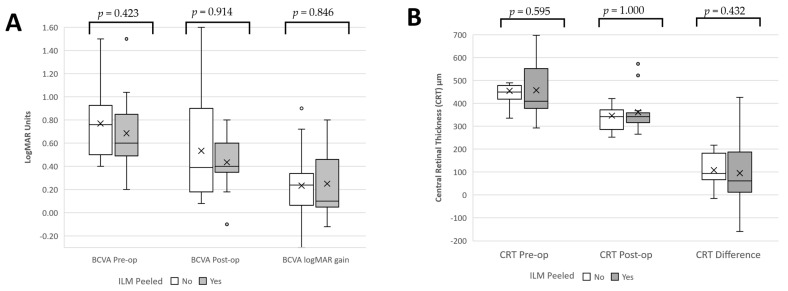
Effect of Internal Limiting Membrane peel on central retinal thickness and visual acuity in uveitic ERM peels at six-months. CRT: Central Retinal Thickness, ILM: Internal Limiting Membrane, ERM: Epiretinal Membrane, BCVA: Best Corrected Visual Acuity. CRT difference = Pre-operative CRT—Post-operative CRT. X denotes mean. Boxplot and whiskers are median and interquartile range. ILM peeling was not found to contribute any difference in (**A**) improvement in CRT or (**B**) logMAR Gain.

Among the subgroup that was treated with combined ILM peeling (*n* = 13), the final mean BCVA improved in eight (62%) eyes and remained stable in five (39%). No difference was detected in logMAR gain between ILM peeling and isolated ERM peeling (*p* = 0.682, Figure 2A). 

### 3.4. Anatomical Outcomes and Treatment 

**Figure 3:**
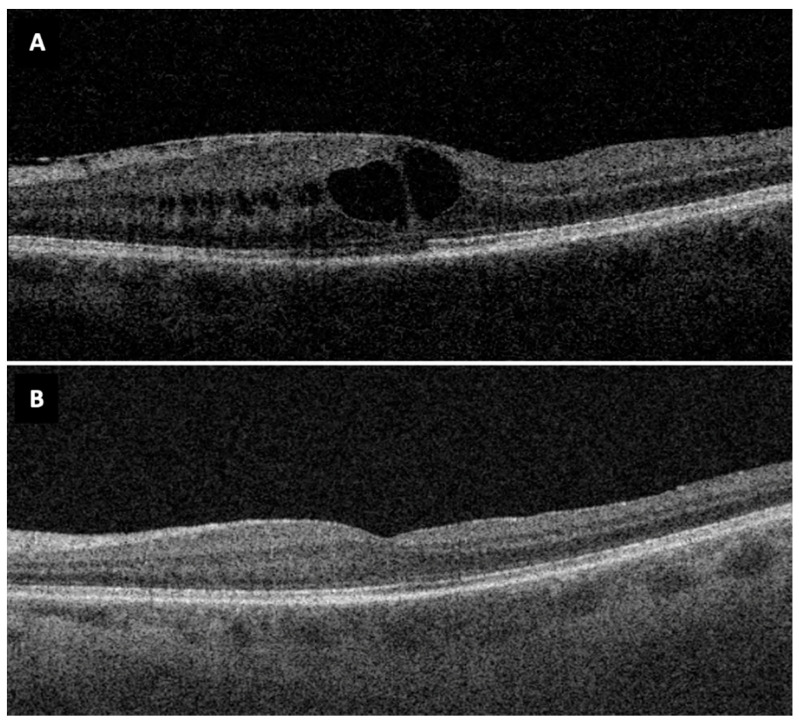
Macular OCT before (**A**) and 6 months after (**B**) vitrectomy and epiretinal membrane peel in a 71-year-old patient diagnosed with chronic anterior uveitis.

At six-month FU, mean CRT decreased significantly from 456 (99) μm to 353 (86) μm (*p* < 0.001, Figure 1B) (Figure 3). Peeling the ILM did not lead to significant differences to changes in CRT (*p* = 0.432, Figure 2B).

The rate of eyes with concomitant CME decreased from 19 (66%) eyes preoperatively to 12 (41%) eyes at six months (*p* = 0.065) and 8 (28%) eyes at final follow-up (*p* = 0.003). We performed subgroup analysis on eyes with residual CME at six-month FU aiming to assess the potential beneficial impact of surgery on logMAR gain and CRT reduction. We found that eyes with persistent postoperative CME did not improve in either of these metrics at six months, but had a significant improvement in mean BCVA (*p* = 0.020) at final FU.

Other postoperative complications included retinal detachment and hypotony (1 (3%), <5 mmHg intraocular pressure), transient secondary ocular hypertension that was controlled with topical eyedrops (2 (7%)), anterior capsular phimosis (1 (3%)), and posterior capsular opacification (1 (3%)). 

### 3.5. Uveitis Status and Therapeutic Regimen

Postoperatively, vitreous inflammation resolved in all three cases. Nine eyes (31%) had AC inflammation at the final FU. Comparing eyes with inflammation involving the anterior versus the posterior segment, we found a significantly better pre-operative BCVA (0.50 (0.17)) in the former compared to the latter (0.84 (0.29), *p* = 0.002). On the other hand, we did not find any differences in final mean BCVA, logMAR gain, mean preoperative and postoperative CRT and CRT-reduction between the two groups; however, this is beyond the scope of this study, and it may not be sufficiently powered to detect these differences. 

**Figure 4:**
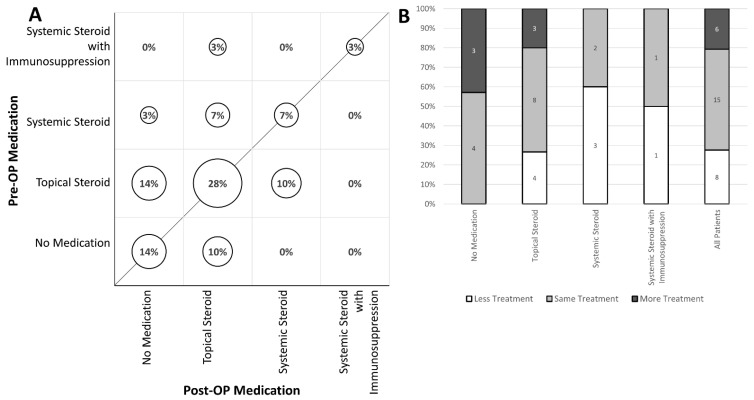
Comparison of Pre- and Post-ERM Surgery Uveitis Medication. (**A**) Bubble plot of medication for all patients. The size of each circle is proportional to the total number of observations and the labels are the percentage of eyes. Above the line represent patients with reduced medication regime post operatively. McNemar-Bowker test *p* = 0.673. (**B**) Overall, 52% of patients were on the same treatment class, 28% had less treatment, and 21% had their treatment increased.

The therapeutic regimens encountered are summarized in a bubble plot showing the change in each treatment class pre- to post-operatively (Figure 4A). Post-operatively, 52% of patients were on the same treatment class, 28% had less treatment and 21% had their treatment increased, relative to their stable pre-operative regime (*p* = 0.673) (Figure 4B). 

Lastly, comparing patient that undergone PPV with active uveitis at the time of surgery to those with inactive uveitis, we did not find any differences in BCVA logMAR gain (*p* = 0.780), CRT reduction (*p* = 0.322) or treatment class post-operatively (*p* = 0.489) between the two groups.

## 4. Discussion

ERMs form due to fibrocellular proliferation and extracellular matrix deposition at the vitreoretinal junction. The contraction of ERMs leads to an increase in CRT. The centrifugal tractional forces are relieved by peeling the ERM, which allows the retina to regain a more normal position [19]. Surgical treatment of idiopathic ERM in symptomatic patients is commonly performed with favourable anatomical and functional results [20]. However, it is also known that the impact of surgery can be different in idiopathic versus secondary ERM, such as diabetic or uveitic ERM [21,22]. In addition, in uveitis ERM concomitant active inflammation and/or CME and/or previous inflammatory macular involvement and residual scarring might act as confounding factors when assessing surgical outcomes [22,23]. Consequently, the role of PPV with ERM peeling in uveitis eyes remains a controversial topic. In this multicentric study, we aimed to evaluate the anatomical and functional outcomes in patients with ERM secondary to uveitis, as well as describe the changes in the therapeutic regimen following surgical intervention. To our knowledge, this series includes the largest number of patients treated for ERMs secondary to uveitis. 

In the light of the crucial role of macular OCT in the assessment, surgical planning, and prognostication of ERM [6], all eyes have been evaluated with complete ophthalmic examination and OCT both preoperatively and postoperatively. We chose to analyse CRT as main anatomical outcome as it has been shown to be related to both preoperative and postoperative VA [24]. We found a significant improvement in both CRT and BCVA across the whole cohort of eyes analysed, with a visual acuity improvement in 66% of cases and CRT reduction in 19 eyes. This is consistent with the favourable anatomical and functional effects of vitrectomy and ERM peel observed in ERMs secondary to uveitis of different aetiologies, such as sarcoidic uveitis [8,11], toxoplasmic retinochoroiditis [25], and pars planitis [10]. It has been shown on a large multivariate analysis that ERM is significantly associated with intermediate, posterior, and panuveitis, and it can independently lead to visual acuity loss in uveitic eyes [3]. In our study, 19 eyes (66%) had a history of either intermediate, posterior, or panuveitis. The association between increased CRT and worse VA in uveitic eyes is still not clear [26,27]. It has been speculated that the stronger traction generated by ERMs leads to a thickening of the central retina and consequent disruption of the ellipsoid zone [27]. The latter is one of the anatomical patterns that have been proposed to correlate lower VA, such as foveal ERM and focal ERM attachment [26]. In our cohort we found that ERM surgery led to a significant reduction in CRT as well as a significant improvement in BCVA.

From a surgical point of view, the role of ILM peeling in PPV for ERM is still debated [28], particularly in case of secondary ERM and/or CME [9,29,30]. Additional ILM removal in PPV for idiopathic ERM was proved to potentially reduce the ERM recurrence rate, but with no influence on the visual outcome and CRT postoperatively [31,32]. Similarly, Wiechens et al. found no difference in the visual outcomes in eyes affected by intermediate uveitis with CME, in absence of ERM, which underwent PPV with or without ILM removal [33]. In our cohort, the peeling of the ILM had no significant impact on CRT reduction and/or logMAR gain in BCVA, consistently with what previously reported by Tanawade et al. [9] Our findings are also similar with the favourable results reported by a recent retrospective study in which all patients were treated with PPV and combined ERM/ILM peeling [22]. Finally, no case of ERM recurrence of ERM was recorded during the follow-up period analyses in this study. 

Another major concern in the surgical planning of eyes with uveitis or history of uveitis, is the activity status of the uveitis and/or the potential risk of reactivation/recrudescence of the uveitis [7]. Surgical intervention in patients with uveitis is always at the risk of flaring otherwise stable uveitis control. On the other hand, PPV itself can be indicated for therapeutic purpose in patients with uveitis for significant media opacities, endophthalmitis, inflammatory control, or structural complications of uveitis, including CME refractory to medical treatment, ERM, and chronic hypotony [34]. In addition, is has been proposed that with vitrectomy, a beneficial effect on uveitis activity may be due to removal of inflammatory matrix in the vitreous and this may result in a reduced need of anti-inflammatory and immunosuppressive therapeutic agents [34]. A significant improvement in VA has been recently reported by Rao et al. [35] after PPV with ERM peeling in patients with uveitis inactive for more than three months before surgery. Whether this improvement is achievable in cases of uveitis activity is still not established. A previous case series of 16 patients with uveitis reported favourable functional results after PPV and ERM peeling even if four of them (25%) had active inflammation preoperatively [9]. In our series, 11 patients had active inflammation at the time of surgery. Interestingly, we found that operating on patients with active uveitis was not detrimental to the surgical outcomes as the presence of active inflammation was not negatively associated with CRT reduction or logMAR gain. This was further reinforced by the analysis of the therapeutic regimen of the patients before and after surgery. Indeed, most patients remained on the same treatment class, a third required less treatment than before surgery, and only about a fifth required a more intensive treatment class, with no patient requiring additional conventional or biologic DMARD. The final beneficial impact of surgery on the therapeutic regimen is a novel finding of this study. So far, controversial results have been reported in terms of changes in therapeutic regimen after PPV in uveitic patients. For instance, a previous case series of patients diagnosed with sarcoidosis reported no additional postoperative OS, despite the initial inflammatory status [8]; whereas, in another study that included only patients with inactive uveitis at surgery, 33% of them needed additional immunosuppression therapy postoperatively [35]. 

Finally, we analysed the impact of surgery on CME associated with ERM. CME is not only an established and common complications of uveitis [36], but also a known complication of PPV itself [37]. All patients were assessed with OCT and, if needed, FA to distinguish between residual cystic spaces mainly associated with the previous ERM-induced traction at the vitreoretinal interface and exudative macular edema, likely to be related with inflammation. Indeed, it has been demonstrated that OCT imaging can be particularly helpful in differentiating between tractional and exudative components of macular edema of different aetiologies [38,39]. We found that PPV with ERM peeling was associated with a significant beneficial impact on CME, as the rate of eyes with concomitant CME decreased from 66% preoperatively to 24% at the last follow-up visit. Moreover, even in eyes with persistent postoperative CME, there was a significant reduction in CRT following surgery. This favourable effect may be linked to both removal of inflammatory mediators in the vitreous [11,40] and removal of the tangential forces acting on the retinal surface [5].

We acknowledge that the present study has some limitations, including a small sample size, the lack of a control arm, the inclusion of ERM of variable duration, and a potential surgeon-related bias regarding the decision to remove ILM. Nevertheless, to date, this is the largest series on the role of vitrectomy in patients with ERM secondary to uveitis from two large tertiary referral centres in the UK. Further prospective studies to have visual acuity, metamorphopsia, and OCT parameters as primary outcomes elements are needed to confirm these results.

## 5. Conclusions

In conclusion, our study showed that PPV with ERM peeling in patients with uveitis was associated with improvement of BCVA, reduction of CRT and resolution of concomitant CME, regardless of combined ILM peeling and/or the presence of active uveitis at the time of surgery. Additionally, although the improvement in the therapeutic regimen after surgery was not significant, it was alongside substantially improved BCVA and CRT reduction. Therefore, PPV with ERM peeling can be beneficial in the management of uveitic ERMs. 

## Figures and Tables

**Table 1:** Preoperative and postoperative uveitis characteristics.

Type, Aetiology	Baseline Tx	Preop Uveitis Status	Postop Active Uveitis	Preop CME	Postop CME	Tx at Final FU	Complications	Preop BCVA	6-Month BCVA	Final BCVA
panuveitis, sclerouveitis, idiopathic	TS + OS	Inactive	Inactive	Yes	Yes	TS + OS	-	0.90	0.40	0.10
anterior uveitis, idiopathic	TS + OS	Inactive	Inactive	Yes	Yes	TS	Unstable IOL, anterior capsule phimosis	0.70	1.00	1.00
posterior uveitis, CMV immune recovery related uveitis	TS + OS	Mild AC inflammation	Mild AC inflammation	Yes	No	TS + OS + IVT ganciclovir	CMV retinitis	1.50	0.70	0.80
posterior uveitis, CMV immune recovery related uveitis	TS	Mild AC inflammation	Mild AC inflammation	Yes	No	TS	-	0.50	0.40	0.40
Panuveitis, toxoplasmosis	TS	Marked AC inflammation	Moderate AC inflammation	N/A	Yes	TS	cataract	1.00	0.10	0.22
Anterior uveitis, idiopathic	TS	Mild AC inflammation	Inactive	Yes	No	TS	-	0.50	0.50	0.20
Intermediate uveitis	TS	Vitritis	Inactive	Yes	Yes	TS	RD, hypotony	0.90	0.94	0.96
sympathetic ophthalmia	TS + OS + IFX	Inactive	Inactive	Yes	Yes	TS + OS + IFX	-	0.80	0.08	0.40
posterior uveitis, sarcoidosis	TS	Mild AC inflammation	Inactive	Yes	No	TS + OS	NVG, occlusive vasculitis	1.00	0.80	0.60
panuveitis, idiopathic	TS	Moderate AC inflammation	Mild AC inflammation	n/a	No	TS	-	1.04	0.40	0.50
chronic panuveitis, idiopathic	TS + OS + MTX	Vitritis	Mild AC inflammation	Yes	No	TS + AZT	-	0.70	0.60	0.30
posterior uveitis, sarcoidosis	TS + OS + MTX	Vitritis	Mild AC inflammation	Yes	Yes	TS + OS + MTX	OHT	0.50	0.40	0.30
Chronic anterior uveitis	TS	Mild AC inflammation	Inactive	Yes	No	TS	OHT	0.40	0.30	0.50
Chronic anterior uveitis	TS	Inactive	Inactive	Yes	No	TS	OHT	0.50	0.20	0.20
Anterior uveitis, HSV	TS	Inactive	Inactive	No	No	TS	-	0.20	−0.10	−0.10
Intermediate uveitis, sarcoidosis	OS	Mild AC inflammation	Mild AC inflammation	Yes	Yes	TS + OS	-	0.76	0.48	0.20
Intermediate uveitis, idiopathic	OS	Inactive	Inactive	Yes	No	OS	-	0.80	0.18	0.20
Panuveitis, idiopathic	TS	Inactive	Mild AC inflammation	No	No	TS	glaucoma	1.50	1.60	NPL
Posterior uveitis, idiopathic	TS	Inactive	Inactive	N/A	No	TS	BRAO	0.48	0.18	0.20
Posterior uveitis, toxocariasis	-	Inactive	Inactive	Yes	Yes	-	-	0.80	0.80	0.80
Chronic anterior uveitis, idiopathic	TS	Inactive	Inactive	No	Yes	TS		0.40	0.30	0.20
Intermediate uveitis, MS	-	Inactive	Inactive	Yes	Yes	-	-	0.70	0.60	0.50
anterior uveitis, presumed sarcoidosis	TS	Inactive	Inactive	No	No	TS	glaucoma	0.50	0.10	0.30
Panuveitis, toxoplasmosis	TS	Inactive	Inactive	No	Yes	TS	glaucoma, PCO	0.60	0.18	0.30
anterior uveitis, sarcoidosis + TB	-	Inactive	Inactive	No	Yes	PF		0.80	0.48	0.90
anterior uveitis, idiopathic	-	Inactive	Inactive	Yes	No	-	-	0.50	0.18	0.20
Intermediate uveitis, idiopathic	-	Inactive	Inactive	Yes	Yes	-	OHT, FTMH	0.48	0.60	0.60
Intermediate uveitis, idiopathic	TS	Inactive	Inactive	Yes	No	-	cataract	0.76	0.90	0.60
posterior uveitis, CMV	-	Inactive	Inactive	Yes	Yes	TS	-	1.00	0.90	1.00

AZT, azathioprine; BRAO, branch retinal artery occlusion; FTMH, full-thickness macular hole; FU, follow-up; IFX, infliximab; IVT, intravitreal; MTX, methotrexate; NVG, neovascular glaucoma; OHT, ocular hypertension; OS, oral steroids; RD, retinal detachment; TS, topical steroids. PF: Prednisolone 1% drops.

## Data Availability

The data presented in this study are available in Table 1.
